# Molecular characterization and functional analysis of the *Schistosoma mekongi* Ca^2+^-dependent cysteine protease (calpain)

**DOI:** 10.1186/s13071-019-3639-9

**Published:** 2019-07-30

**Authors:** Salisa Chaimon, Yanin Limpanont, Onrapak Reamtong, Sumate Ampawong, Orawan Phuphisut, Phiraphol Chusongsang, Jiraporn Ruangsittichai, Usa Boonyuen, Dorn Watthanakulpanich, Anthony J. O’Donoghue, Conor R. Caffrey, Poom Adisakwattana

**Affiliations:** 1 Department of Helminthology, Faculty of Tropical Medicine, Mahidol University, Bangkok, 10400 Thailand; 2 Department of Social and Environmental Medicine, Faculty of Tropical Medicine, Mahidol University, Bangkok, 10400 Thailand; 3 Department of Molecular Tropical Medicine and Genetics, Faculty of Tropical Medicine, Mahidol University, Bangkok, 10400 Thailand; 4 Department of Tropical Pathology, Faculty of Tropical Medicine, Mahidol University, Bangkok, 10400 Thailand; 5 Department of Medical Entomology, Faculty of Tropical Medicine, Mahidol University, Bangkok, 10400 Thailand; 6 Center for Discovery and Innovation in Parasitic Diseases, Skaggs School of Pharmacy and Pharmaceutical Sciences, University of California San Diego, La Jolla, San Diego, California USA

**Keywords:** *Schistosoma mekongi*, Schistosomiasis, Calcium-dependent cysteine protease, Calpain, Drug and vaccine development

## Abstract

**Background:**

*Schistosoma mekongi*, which causes schistosomiasis in humans, is an important public health issue in Southeast Asia. Treatment with praziquantel is the primary method of control but emergence of praziquantel resistance requires the development of alternative drugs and vaccines. Calcium-dependent cysteine protease (calpain) is a novel vaccine candidate that has been studied in *S. mansoni*, *S. japonicum*, and protozoans including malaria, leishmania and trypanosomes. However, limited information is available on the properties and functions of calpain in other *Schistosoma* spp., including *S. mekongi*. In this study, we functionally characterized calpain 1 of *S. mekongi* (SmeCalp1).

**Results:**

Calpain 1 of *S. mekongi* was obtained from transcriptomic analysis of *S. mekongi*; it had the highest expression level of all isoforms tested and was predominantly expressed in the adult male. SmeCalp1 cDNA is 2274 bp long and encodes 758 amino acids, with 85% to 90% homology with calpains in other *Schistosoma* species. Recombinant SmeCalp1 (rSmeCalp1), with a molecular weight of approximately 86.7 kDa, was expressed in bacteria and stimulated a marked antibody response in mice. Native SmeCalp1 was detected in crude worm extract and excretory-secretory product, and it was mainly localized in the tegument of the adult male; less signal was detected in the adult female worm. Thus, SmeCalp1 may play a role in surface membrane synthesis or host–parasite interaction. We assessed the protease activity of rSmeCalp1 and demonstrated that rSmeCalp1 could cleave the calpain substrate *N*-succinyl-Leu-Leu-Val-Tyr-7-amino-4-methylcoumarin, that was inhibited by calpain inhibitors (MDL28170 and E64c). Additionally, rSmeCalp1 could degrade the biological substrates fibronectin (blood clotting protein) and human complement C3, indicating important roles in the intravascular system and in host immune evasion.

**Conclusions:**

SmeCalp1 is expressed on the tegumental surface of the parasite and can cleave host defense molecules; thus, it might participate in growth, development and survival during the entire life-cycle of *S. mekongi*. Information on the properties and functions of SmeCalp1 reported herein will be advantageous in the development of effective drugs and vaccines against *S. mekongi* and other schistosomes.

**Electronic supplementary material:**

The online version of this article (10.1186/s13071-019-3639-9) contains supplementary material, which is available to authorized users.

## Background

Cysteine proteases presented in all living organisms have been classified into 121 families [1]. In parasites, cysteine proteases are mainly divided into two clans: clan CA proteases are further divided into family C1 (cathepsin B, L, F) and family C2 (calpain-like) and clan CD compose of family C13 (asparaginyl endopeptidase, legumain-like) and C14 (caspase-like) [1]. Calpains, calcium-dependent cysteine proteases, are categorized into cysteine protease clan CA, family C2 (MEROPS database) [1]. These proteases are ubiquitous, being found in bacteria, fungi, plants and animals. Classical calpains are composed of four major domains: the N-terminal prodomain, protease core domain, the C2 domain-like Ca^2+^-binding domain and the EF-hand Ca^2+^ binding domain. Calpains play important roles in various biological processes such as signal transduction, cell morphogenesis, cytoskeletal remodeling, cell cycle regulation, vesicular trafficking, cell differentiation, apoptosis and necrosis [2–8].

In parasitic helminths, calpain 1 and 2 of *Schistosoma mansoni* and calpain 1 of *S. japonicum* have been identified, characterized and shown to be predominantly expressed on the tegument, surface syncytial epithelium and in the underlying musculature of adult parasites [9–11]. These localizations may indicate their roles in host–parasite interaction, immune evasion and membrane turnover processes [9–13]. In *S. japonicum*, calpain was found in the excretory pore, secretory gland and secretory product of cercariae, which suggests a role in penetration of host tissue and migration [11, 13]. In a recent study of *S. mansoni* calpains (SmCalps), native SmCalps (SmCalp1, SmCalp2, or both) could cleave the host blood clotting factor fibronectin, which implies that SmCalps may protect against blood clot formation around worms living in the blood circulation [10].

Calpain has recently been proposed and intensively studied as a promising target for vaccine development to prevent and control schistosomiasis. Immunizing mice with a *S. mansoni* calpain (Sm-p80) DNA vaccine demonstrated 30–60% and 23–84% reductions in worm burden and egg fecundity, respectively [14–16]. Evaluation of Sm-p80 vaccine efficacy in baboons demonstrated a 38% reduction of hepatic egg burden and a > 50% reduction in egg load in the small and large intestines. Moreover, vaccination interfered with egg maturation and miracidia hatching, with a significant reduction in the hatching rate of eggs obtained from the small and large intestines (approximately 50–70%) [17]. Mice vaccinated with recombinant *S. japonicum* calpain (rSjCALP) showed decreased worm burden (41.2%), egg fecundity and pathological severity [18]. Treatment of *S. japonicum* schistosomula with rSjCALP-immunized sera before incubation with murine peritoneal exudate cells *in vitro* showed tight adhesion of peritoneal exudate cells around the schistosomula and antibody-dependent cell-mediated cytotoxicity [13].

Although *Schistosoma* calpains have been identified and evaluated as vaccine candidates for nearly 30 years, information regarding their properties and functions remains limited. Moreover, the available literature and databases have described only calpains derived from *S. mansoni* and *S. japonicum*. Thus, the functions of calpain need to be intensively identified and characterized in other human *Schistosoma* species to develop pan-inhibitor and pan-vaccine against all species causing schistosomiasis in both humans and animals. In this study, we identified and functionally characterized calpain of *S. mekongi*, the causative agent of Mekong schistosomiasis that is endemic in the Khong island areas of the Lao People’s Democratic Republic (Lao PDR) and Cambodia. Although *S. mekongi* occurs in a small, restricted area, many people (approximately 140,000) are at risk of infection [19–21]. Furthermore, cases of Mekong schistosomiasis have occurred not only in local people, but also in travelers to Lao PDR and Cambodia [22]. The aim of this study was to obtain the full-length coding sequence of *S. mekongi* calpain 1 (SmeCalp1) from an adult *S. mekongi* transcriptome library [23] and then predict the molecular properties using bioinformatics analysis. The recombinant SmeCalp1 protein was heterologously expressed in *Escherichia coli* and used for further molecular characterization. We determined the location of SmeCalp1 in parasite tissue by immunohistochemistry and immunogold electron microscopy. We also evaluated the biological functions by hydrolysis of fluorogenic peptides and biological substrates.

## Methods

### Maintaining *S. mekongi*

Different developmental stages of *S. mekongi* were provided by the Applied Malacology Laboratory, Department of Social and Environmental Medicine, Faculty of Tropical Medicine, Mahidol University, Bangkok, Thailand. The life-cycle was maintained in *Neotricular aperta* snails and ICR mice. Adult worms were obtained from mice at 8 weeks post-infection using the perfusion technique [24]. Eggs were obtained by homogenizing infected intestines and livers in normal saline solution and then filtering to remove tissue contamination [25]. Miracidia were collected from eggs by light induction, as described previously [26]. Cercariae were shed from the snails at approximately 6 weeks post-infection by light induction and then transferred into a conical tube before centrifugation at 6000×*g* at 4 °C for 20 min. The schistosomules were prepared by *in vitro* transformation of cercariae using a 22-gauge, double-ended, Luer-Lok emulsifying needle attached to a 20-ml syringe in each side, as described previously [27]. All developmental stages were kept at − 80 °C for further studies.

### Bioinformatics analysis

Different isoforms of full length SmeCalp were obtained from the transcriptomic database of adult *S. mekongi* [23]. The transcription levels of SmeCalp isoforms were compared between adult male and female parasites as well as among isoforms. The mRNA sequences of SmeCalp isoforms were used to design specific primers to analyze their transcription level using SYBR real-time reverse transcription (RT)-PCR as described below. The sequences and accession numbers used in this study are provided in Additional file 1: Table S1.

A full-length coding sequence of SmeCalp1 (GenBank: MK610444) was selected because it was the most expressed isoform in adult male compared with others. The deduced amino acid sequence of SmeCalp1 was submitted to BLASTP to determine the percentage homology and identify closely related orthologs. Additionally, the amino acid sequence was used to predict properties, including classical secretory pathway (signal peptide; SignalP 4.1 Server) [28], non-classical secretory pathway (SecretomeP 2.0 server) [29], transmembrane regions (TMpred) [30] and *N*-,*O*-glycosylation sites (NetNGlyc 1.0 or NetOGlyc 4.0) [31, 32]. The conserved motifs and consensus residues of SmeCalp1 were compared with orthologs using Clustal Omega [33]. The phylogenetic tree was constructed using the maximum likelihood method (1000 bootstrap replication) using the program MEGA7 [34] to analyze evolutionary relationships among SmeCalp1 and its orthologs. All sequences used in this study are detailed in Additional file 2: Table S2.

The 2-dimensional structure of SmeCalp1 was predicted using the SABLE program [35] and the structural image created using the Polyview method [36]. A 3-dimensional structure simulation of SmeCalp1 was performed by SwissModel [37] using template crystal structure of human m-calpain form II (PDB ID: 1KFU); the structural image was generated using iCn3D [38].

### Construction of recombinant SmeCalp1

Total RNA was extracted from adult *S. mekongi* using TRIzol reagent (Invitrogen, Carlsbad, CA, USA) following the manufacturer’s instructions. The total RNA was subsequently treated with DNase (1 U of DNase/µg of total RNA) to remove any contaminating genomic DNA and converted to first-strand cDNA using reverse transcriptase (Thermo Fisher Scientific Inc., Vilnius, Lithuania). The full-length SmeCalp1 cDNA was amplified from the first-strand cDNA template using the following primers: forward (Fw) (5′-GGA TCC GAT GGG ACG AAT ACA AAT TGT ATA TT-3′) and reverse (Rv) (5′-CTC GAG AAT GTA AAC GGC AAA GCG TAG-3′) which incorporated *Bam*HI and *Xho*I restriction sites (underlined), respectively. The PCR product was examined using 1% agarose gel electrophoresis. The PCR product was ligated into the pGEM-T Easy vector (Promega Corporation, Madison, WI, USA) and subcloned into the pET20b^+^ prokaryotic expression vector.

### Expression, purification and refolding of recombinant SmeCalp1

The recombinant plasmid pET20b^+^-SmeCalp1 was transformed into *E. coli* BL21 (DE3) pLysS by the heat-shock transformation method [39]. Protein expression was induced by adding isopropyl β-d-1-thiogalactopyranoside (IPTG, Thermo Fisher Scientific Inc.) to a final concentration of 1 mM and then culturing for 4 h. Cells were harvested by centrifugation at 6000×*g* at 4 °C for 30 min and resuspended in lysis buffer (6 M Gu-HCl, 50 mM NaH_2_PO_4_ and 300 mM NaCl, pH 8.0) for 60 min. The suspension were centrifuged at 14,000×*g* at room temperature (RT) for 30 min and the supernatant was incubated with Talon Metal Affinity Resin (Clontech Laboratories Inc., Mountain View, CA, USA) at RT for 1 h, followed by loading the resin into a Talon 2 ml Disposable Gravity Column (Clontech Laboratories Inc.). Recombinant SmeCalp1 (rSmeCalp1) was purified as described previously [26]. Purified rSmeCalp1 was analyzed by sodium dodecyl sulfate polyacrylamide gel electrophoresis (SDS-PAGE) and expression was confirmed by western blot analysis reacting with mouse anti-His tag antibody (BioLegend, San Diego, CA, USA) and mass spectrometry. rSmeCalp1 was stepwise dialyzed against 1× PBS to remove excess urea before immunizing mice for production of polyclonal antibody.

To prepare rSmeCalp1 for activity assay, purified rSmeCalp1 was refolded as described previously with some modification [40, 41]. Briefly, purified rSmeCalp1 was rapidly diluted 20-fold in refolding buffer (20 mM Tris-HCl; pH 7.5, 10 mM reduced glutathione, 1 mM oxidized glutathione, 0.7 M l-arginine and 10% glycerol) and then incubated at 4 °C for 24 h. On the following day, the protein was concentrated 20-fold using an Amicon stirred cell (EMD Millipore Corporation, Billerica, MA, USA) through an Ultracel 10 kDa ultrafiltration disc (EMD Millipore), and buffer was exchanged with 20 mM Tris-HCl, pH 7.0, with three buffer exchanges at 4 °C. The refolded rSmeCalp1 was concentrated and used for measurement of enzymatic activity.

### Stage-specific expression of SmeCalp1 isoforms

Total RNA was extracted from eggs, miracidia, cercariae and adult males and females of *S. mekongi*; then, contaminating genomic DNA was removed using DNase and RNA was converted to first-strand cDNA by reverse transcriptase. The levels of SmeCalp1 transcripts in different developmental stages were quantified using SYBR Green real-time RT-PCR. The experiment was performed in triplicate in a final volume of 15 µl by mixing 2 µl of first-strand cDNA with 1× iTaq Universal SYBR Green master mix (Bio-Rad Laboratories Inc., Hercules, CA, USA) and 100 nM each of Fw and Rv primers (Additional file 1: Table S1). 18S RNA was used as an internal control, as described previously [42, 43]. Amplification was performed using the MasterCycler Real-Time PCR system (Realplex^4^, Eppendorf, Hamburg, Germany) with pre-incubation at 95 °C for 5 min, followed by 40 cycles of 95 °C for 15 s and 60 °C for 1 min. Melting curve analysis was performed at 65 to 95 °C. The transcription level of SmeCalp1 in each stage was normalized to the expression level of 18S RNA using the formula of 2^−ΔCt^. Relative arbitrary units (A.U.) of SmeCalp1 were compared across all developmental stages.

### Production of mouse anti-rSmeCalp1 polyclonal antibody

Mice at 6 to 8 weeks of age were immunized four times at 2-week intervals by intraperitoneal injection. Before the first injection, pre-immunization sera were collected and stored at − 20 °C. A total of 80 μg of purified rSmeCalp1 containing an equal volume of Imject Alum (Thermo Fisher Scientific Inc.) was injected into mice as the primary injection. Then, 2, 4 and 6 weeks later, the mice were injected with 40 μg of rSmeCalp1 as the second, third and fourth injections, respectively. Blood was collected 1 week after the fourth injection and serum was obtained and stored at − 20 °C until use. The specific antibody titer against rSmeCalp1in each mouse was determined using indirect ELISA, as previously published [44, 45].

### Preparation of parasite antigens

Crude worm antigen (CWA) was prepared by homogenizing adult worms in phosphate buffered saline (PBS) containing 1% Triton X-100. After homogenization, the homogenate was sonicated on ice at an amplitude of 30% with a 9-s on/off pulse using an ultrasonic processor (Vibra-cell, Sonics, Newtown, CT, USA). The CWA was centrifuged at 12,000×*g* at 4 °C for 30 min and the clear supernatant was transferred into several microfuge tubes, followed by storage at − 80 °C until use. Excretory-secretory (ES) product was prepared from cultured adult worms in RPMI-1640, as described previously [26]. The protein concentration was measured using Coomassie Plus Protein Assay Reagent Kit (Thermo Fisher Scientific Inc.) according to the manufacturer’s instructions.

### Immunological detection

CWA, ES and rSmeCalp1 of *S. mekongi* and CWA of *S. mansoni* and *S. japonicum* were size-separated by SDS-PAGE and electrically transferred onto polyvinyl difluoride (PVDF) membranes (Pall Corporation, Port Washington, NY, USA). Adult *S. mansoni* and *S. japonicum* were gifted by the Applied Malacology Laboratory, Department of Social and Environmental Medicine, Faculty of Tropical Medicine, Mahidol University, Bangkok, Thailand. The membranes were washed with 1× PBS containing 0.05% Tween 20 (PBST) and blocked with 5% skimmed milk at RT for 1 h. To detect native SmeCalp1, the membrane was washed three times with PBST and incubated with a 1:2000 dilution of mouse anti-rSmeCalp1 polyclonal antibody (pAb) at 4 °C overnight. After washing three times with PBST, the membrane was incubated with 1:2000 horseradish peroxidase (HRP)-conjugated goat anti-mouse IgG (Southern Biotech, Birmingham, AL, USA) at RT for 1 h. The bands were visualized by adding 2, 6*-*dichloroindophenol sodium salt hydrate (DCIP) substrate (Sigma-Aldrich Co., St. Louis, MO, USA).

### Immunolocalization of SmeCalp1 in parasite tissue

Fresh adult worms were immediately fixed with 4% paraformaldehyde in 1× PBS and dehydrated before embedding in paraffin. Paraffin-embedded specimens were cut into 5-μm sections, followed by deparaffinization, rehydration, retrieval of antigenic epitopes by boiling in antigen retrieval solution (10 mM sodium citrate buffer, pH 6.0, and 1 mM EDTA, pH 8.0) and inactivation of endogenous peroxidase, as previously described [46]. Sections were blocked with blocking solution (10% fetal bovine serum in 1× PBS, pH 7.4) and subsequently incubated with mouse anti-rSmeCalp1 (1:500) or pre-immune sera (1:500) at 4 °C overnight and then conjugated with HRP-conjugated goat anti-mouse IgG (1:1000; Southern Biotech). Color was developed using the AEC staining kit (Sigma-Aldrich Co.) according to the manufacturer’s instructions, and sections were subsequently examined with a light microscope.

The ultrastructural localization of SmeCalp1 was determined using immunogold-labeled transmission electron microscopy. In detail, fresh adult worms were fixed in 2.5% glutaraldehyde in 0.1 M sucrose phosphate buffer, pH 7.4, for 1 h, and the fixed worms were further prepared to obtain sections 90–100 nm thick, as described previously [47]. To perform immunogold labeling, the sections were incubated in 50 mM glycine in PBS, pH 7.4, to quench free aldehyde. Then, non-specific binding was blocked with 5% bovine serum albumin in PBS, pH 7.4, and the sections were incubated with 1:50 mouse anti-rSmeCalp1 for 1 h followed by 1:50 goat anti-mouse IgG conjugated with 3 to 5 nm gold (G7402-.4ML; Sigma Chemical Co., St. Louis, MO) for 1 h. After washing the sections with 0.1% bovine serum albumin in PBS and distilled water, the nanogold particle signaling was enhanced using a silver enhancement kit (Aurion R-Gent SE-EM kit, 25521; EMS, Hatfield, PA) according to the manufacturer’s instructions, and the sections were stained with uranyl acetate and lead citrate [47]. Sections were visualized under a transmission electron microscope (HT7700, Hitachi Ltd., Tokyo, Japan).

### Determination of calpain activity

Enzymatic activity of rSmeCalp1 was measured by incubating the protein with calpain fluorogenic substrate, 40 µM *N*-succinyl-Leu-Leu-Val-Try-7-amino-4-methylcoumarin (AMC) (Sigma-Aldrich Co.), in an assay buffer (100 mM Tris-HCl pH 7.5, 100 mM NaCl, 5 mM CaC1_2_ and 1 mM dithiothreitol) in a total volume of 100 µl. For the inhibitory assay, rSmeCalp1 was pre-incubated with calpain inhibitor (100 µM MDL28170; EDM Millipore Corp., Darmstadt, Germany) and other protease inhibitors (100 µM E64, 100 µM E64c, 100 µM phenylmethylsulfonyl fluoride (PMSF), 1 mg/ml pepstatin, 5 mM EDTA and 1 mM 1,10-phenanthroline; Sigma-Aldrich Co.) at 25 °C for 1 h before adding the substrate. Enzymatic activity was measured by monitoring the release of free fluorescence (AMC) upon hydrolysis of substrate at 37 °C for 30 to 60 min using a fluorometer (Synergy^H1^ Hybrid Reader, BioTek, Winooski, VT, USA) with excitation at 360 nm and emission at 460 nm [48]. Recombinant mouse dihydrofolate reductase, rmDHFR, was used as an irrelevant protein control. To determine the pH optimum, rSmeCalp1 was incubated with substrate at various pH buffers ranging from pH 4.5 to 9.5 (100 mM sodium acetate for pH 4.5 and 5.5; 100 mM Tris-HCl for pH 6.5, 7.5, 8.5 and 9.5). According to previous studies, the activity of calpain depends on calcium concentration. Therefore, we assayed the activity of rSmeCalp1 in buffers with different calcium concentrations (0, 10 µM, 50 µM, 0.1 mM, 0.5 mM, 1 mM, 5 mM and 10 mM) as reported previously [11, 49, 50].

To determine the proteolytic activity of rSmeCalp1 against biological substrates, different substrates, including 20 µg of human hemoglobin (Sigma-Aldrich Co.), 20 µg of mouse IgG (Invitrogen, Carlsbad, CA, USA), 20 µg of bovine albumin (USB Corp., Cleveland, OH, USA), 20 µg of human plasma fibronectin (EDM Millipore Corp.), 20 µg of human complement C1q or 20 µg of C3 (EDM Millipore Corp.), were added to 100 µl of assay buffer (100 mM Tris-HCl, pH 7.5, 100 mM NaCl, 5 mM CaC1_2_) and incubated at 37 °C. Subsequently, small aliquots were collected at various time intervals. For the inhibitory assay, rSmeCalp1 was pre-incubated with E64 inhibitor (1 mM; Sigma-Aldrich Co.) at 25 °C for 4 h before adding the substrate. The reaction was terminated by adding 3× SDS-PAGE sample buffer and then heating at 100 °C for 5 min. The SDS-PAGE gel was visualized by staining with Coomassie Brilliant Blue solution (Serva, Heidelberg, Germany).

## Results

### Sequencing and bioinformatics

**Fig. 1:**
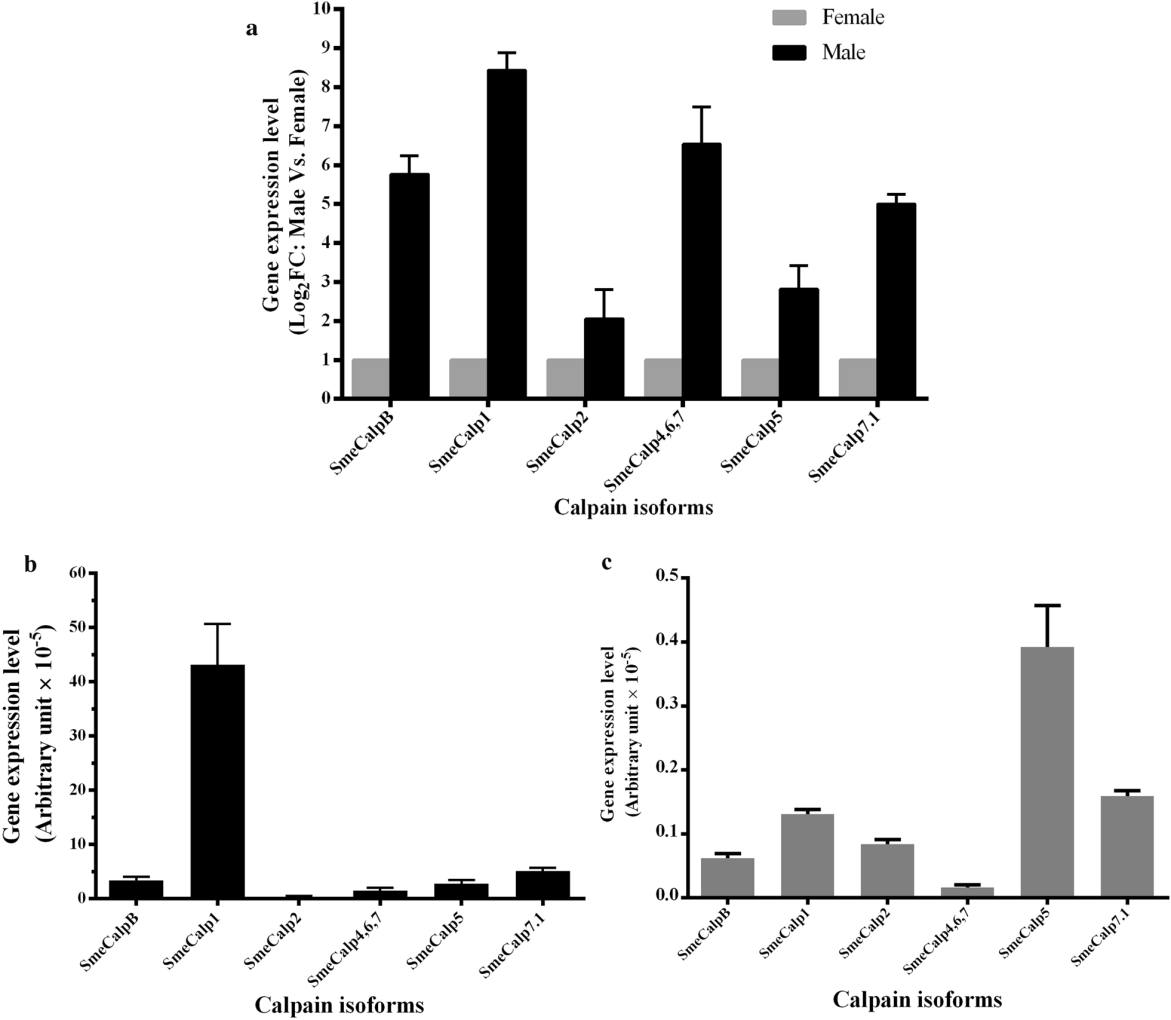
Gene expression level of calpain isoforms in adult male and female *Schistosoma mekongi*. **a** Gene expression level of calpain isoforms was determined using SYBR real-time RT-PCR, and the relative expression level of each isoform between adult males and females was calculated as Log_2_ fold-change (Log_2_FC). Comparison of gene expression level (in arbitrary units) of calpain isoform in **b** adult male and **c** adult female. Error bars show standard deviations

mRNA sequences of calpains obtained from the transcriptomic databases of adult male and female *S. mekongi* [23] comprised 6 isoforms, including SmeCalpB, SmeCalp1, SmeCalp2, SmeCalp4,6,7, SmeCalp5 and SmeCalp7.1 (Additional file 1: Table S1). The transcription level of each isoform was determined in adult male and female *S. mekongi* using SYBR real-time RT-PCR and the results indicated that all SmeCalp isoforms were predominantly expressed in adult males. Compared with other isoforms, transcription of SmeCalp1 showed the greatest difference between adult males and females (Fig. 1a). SmeCalp1 was also transcribed at the highest level of all isoforms expressed in adult males (Fig. 1b), while SmeCalp5 was predominantly expressed in adult females (Fig. 1c). The deduced amino acid sequence of SmeCalp1 was translated and the National Center for Biotechnology Information (NCBI) database was searched using BLASTP for homologous sequences. SmeCalp1 was found to be closely related to calpain 1 of *S. mansoni* (SmCalp1.3) and *S. japonicum* (Sj-CCalp1), which have also been recently investigated for potential vaccine development against schistosomiasis [13, 18, 51, 52]. Therefore, we selected SmeCalp1 for further study to characterize its molecular properties and functions. Sequence analysis of SmeCalp1 demonstrated that its coding sequence consisted of 2274 nucleotides, encoding a protein of 758 amino acid residues. The predicted molecular mass and isoelectric point of SmeCalp1 were approximately 86.7 kDa and 5.26, respectively.

We predicted the potential secretion of SmeCalp1 regarding the classical (SignalP 4.1) and non-classical (SecretomeP 2.0) secretory pathways, and the results suggested that SmeCalp1 does not contain both an N-terminal signal peptide and a non-classical secretory signal. However, SmeCalp1 contains a transmembrane helix at the N-terminus, located at residues 149 to 170 (TM score = 1399; cut-off score ≥ 500). Prediction of disulfide bond formation in SmeCalp1 demonstrated that this protease has 10 cysteine residues at residues C_154_, C_243_, C_290_, C_294_, C_387_, C_394_, C_431_, C_460_, C_633_ and C_722_. Three potential *N*-glycosylation sites are predicted in SmeCalp1 at residues N_13_, N_108_ and N_616_, and 5 potential *O*-glycosylation sites are predicted at residues S_9_, S_15_, S_30_, T_40_ and T_66_.

**Fig. 2:**
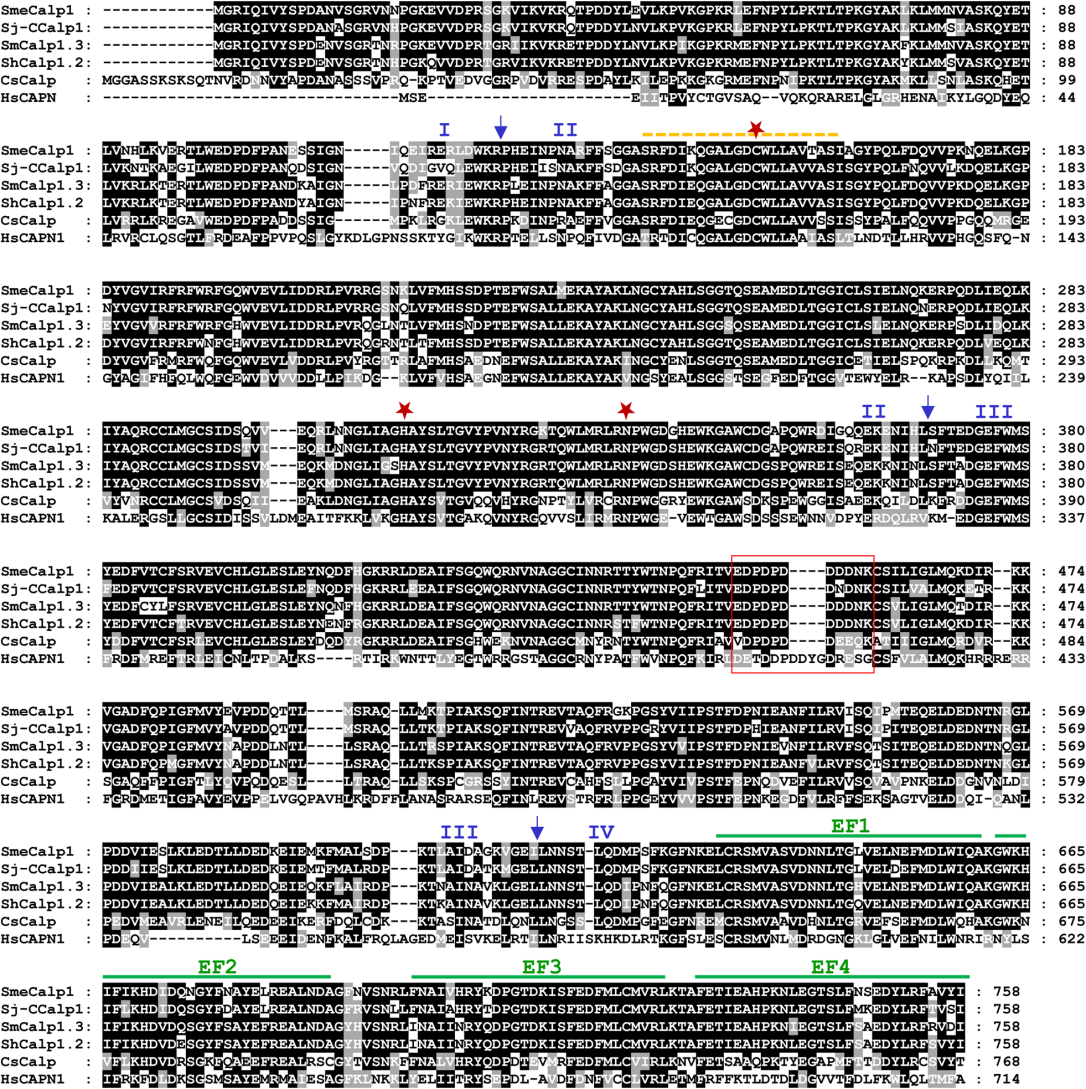
Multiple sequence alignment of SmeCalp1 sequence with orthologs. The deduced amino acid sequence of calpain 1 homologs *Schistosoma mekongi* (SmeCalp1), *S. japonicum-*Chinese strain (Sj-CCalp1), *S. mansoni* (SmCalp1) and *S. haematobium* (ShCalp1) were aligned using Clustal Omega program. The identical and similar amino acids are shaded in black and gray, respectively. Gaps (−) are introduced to optimize homology. The arrows indicate the regions of domains I, II, III and IV. The cysteine protease active site is indicated by yellow dashed overline. The predicted catalytic triad cysteine (C154), histidine (H313) and asparagine (N337) is indicated by stars. Red box indicates the putative Ca^2+^-binding acidic loop (E-E/D-X-D-D/E-X-D-D/E-D-G-X). The four EF-hand motifs (EF1 to 4) are indicated by green overlines. The GenBank accession numbers of the sequences are provided in Additional file 2: Table S2

Multiple sequence alignment was analyzed to identify conserved motifs and consensus residues of the calpain family in SmeCalp1 by global sequence comparison with calpain 1 orthologs found in *S. japonicum*, *S. mansoni*, *S. haematobium*, *Clonorchis sinensis* and *Homo sapiens.* The results demonstrated that the amino acid sequence of SmeCalp1 is highly conserved with those of calpain 1 in other *Schistosoma* spp. (85–90%) but to a lesser degree with *H. sapiens* (5.88%). SmeCalp1 was composed of four domains: I (N-terminal domain: 1–125); II (protease core domain: 126–369); III (C2 domain-like: 370–613); and IV (EF-hand domain: 614–758). The catalytic triad of SmeCalp1 identified at Cys_145_, His_313_ and Asn_337_ was located in domain II, which is conserved in the calpain 1 group (Fig. 2). Four conserved calcium-binding EF-hand motifs (EF1–EF4) were identified at positions 632–660, 662–690, 698–726 and 730–758, respectively (Fig. 2).

**Fig. 3:**
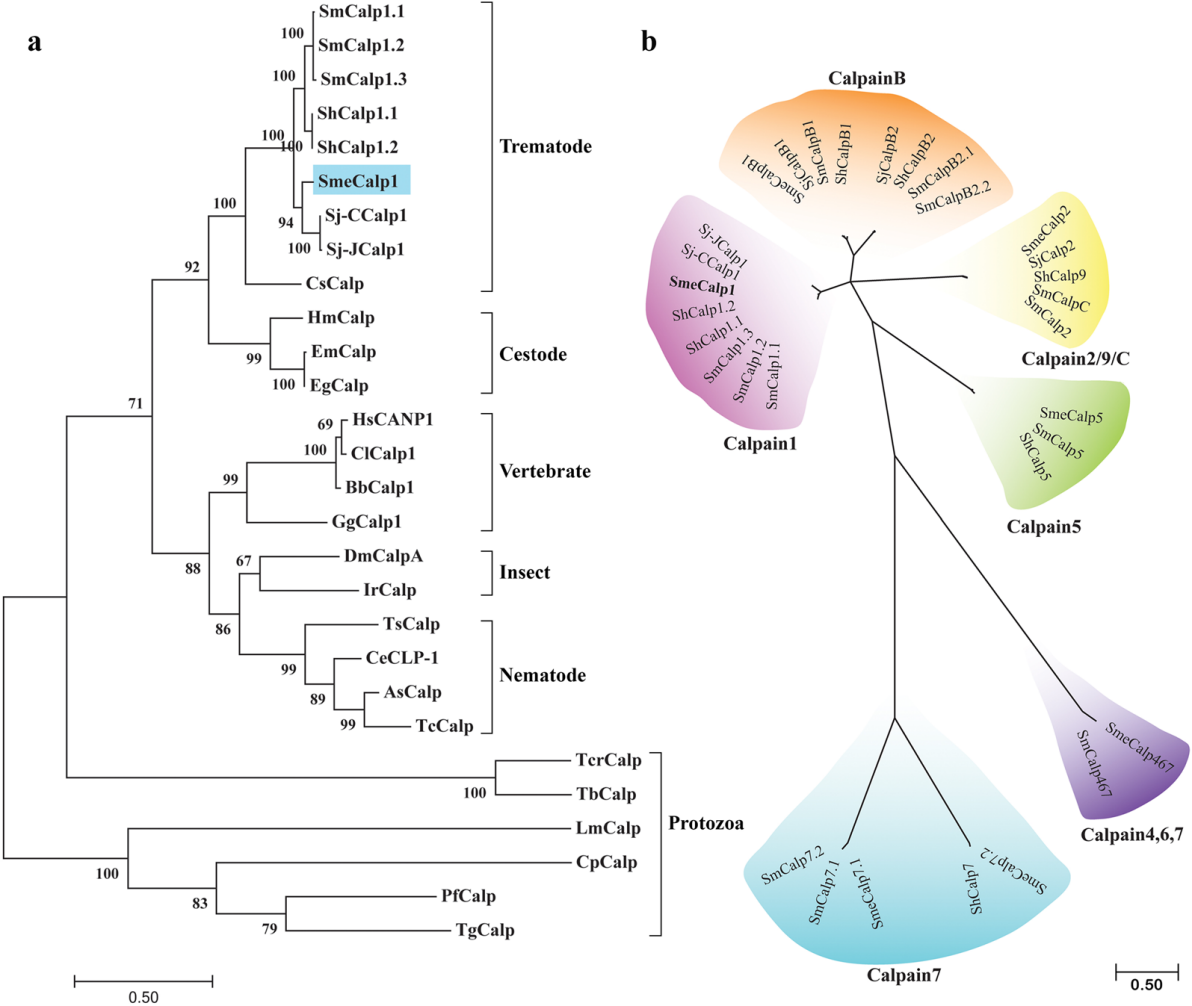
Phylogenetic analysis of the calpains. **a** A rooted tree comparison of SmeCalp1 with other calpain 1 orthologs found in trematodes, cestodes, nematodes, protozoans, insects and vertebrates. **b** An unrooted tree comparison of calpain isoforms identified in human *Schistosoma* spp. The trees were constructed with maximum likelihood method using MEGA7 program, 1000 bootstrap replicates. Numbers at the nodes indicate the proportion of bootstraps. The abbreviations used and GenBank accession numbers are provided in Additional file 2: Table S2

A phylogenetic tree was constructed using 28 calpain orthologs from trematodes, nematodes, cestodes, protozoa, insects and vertebrates. The tree showed that SmeCalp1 was clustered in a trematode clade and was most closely related to other schistosomes, especially Sj-CCalp1. The liver fluke, *C. sinensis*, was located in the same trematode clade but separated in a different branch. The trematode clade was closely related to the cestode clade, both of which belong to phylum Platyhelminthes. Moreover, the phylogenetic tree indicated that SmeCalp1 and the trematode clade were distinctly separated from nematode, insect and vertebrate calpains. Protozoan calpains were located furthest from other clades in the tree (Fig. 3a). A phylogenetic tree of calpain isoforms in genus *Schistosoma* was constructed to determine the diversity of calpains within this genus. The unrooted tree revealed that schistosome calpains could be clustered into 6 major classes: calpain B; calpain 1; calpain 4, 6, 7; calpain 5; calpain 7; and calpain 2/9/C. The results confirmed that SmeCalp1 was classified into the same group as calpain 1 of *S. haematobium*, *S. japonicum* and *S. mansoni*. The other five calpain isoforms of *S. mekongi* were classified into different calpain groups (Fig. 3b).

### *In silico* structure modeling of SmeCalp1

The secondary structure of SmeCalp1 was composed of approximately 36% alpha and other helices, 19% beta strands or bridges and 45% coils. At the C-terminus, a signature EF-hand structure was predicted (helix-loop-helix) where Ca^2+^ bonds were located (Additional file 3: Figure S1a). The tertiary structure of inactive SmeCalp1 (Ca^2+^ free) was predicted using the crystal structure of human m-calpain form II (PDB ID: 1KFU) as a template. The 3-dimensional structure consisted of four domains including domain I, N-terminal domain; domain II separated into protease core domain 1 (PC1) and 2 (PC2); domain III, C2 domain-like (C2L), containing eight antiparallel β-strands (β-sandwich structure); and domain IV, EF-hand domain (EF), the Ca^2+^ binding domain containing four EF-hand motifs (Additional file 3: Figure S1b). In the protease core domain (domain II), PC1 and PC2 fold and interact with each other to create the active site cleft, which is necessary for proteolytic activity and inhibition. The EF-hand motifs found at EF domain (domain IV) contain a helix-loop-helix topology, where calcium ions (Ca^2+^) bind to ligands in the loop.

### Expression and purification of rSmeCalp1

**Fig. 4:**
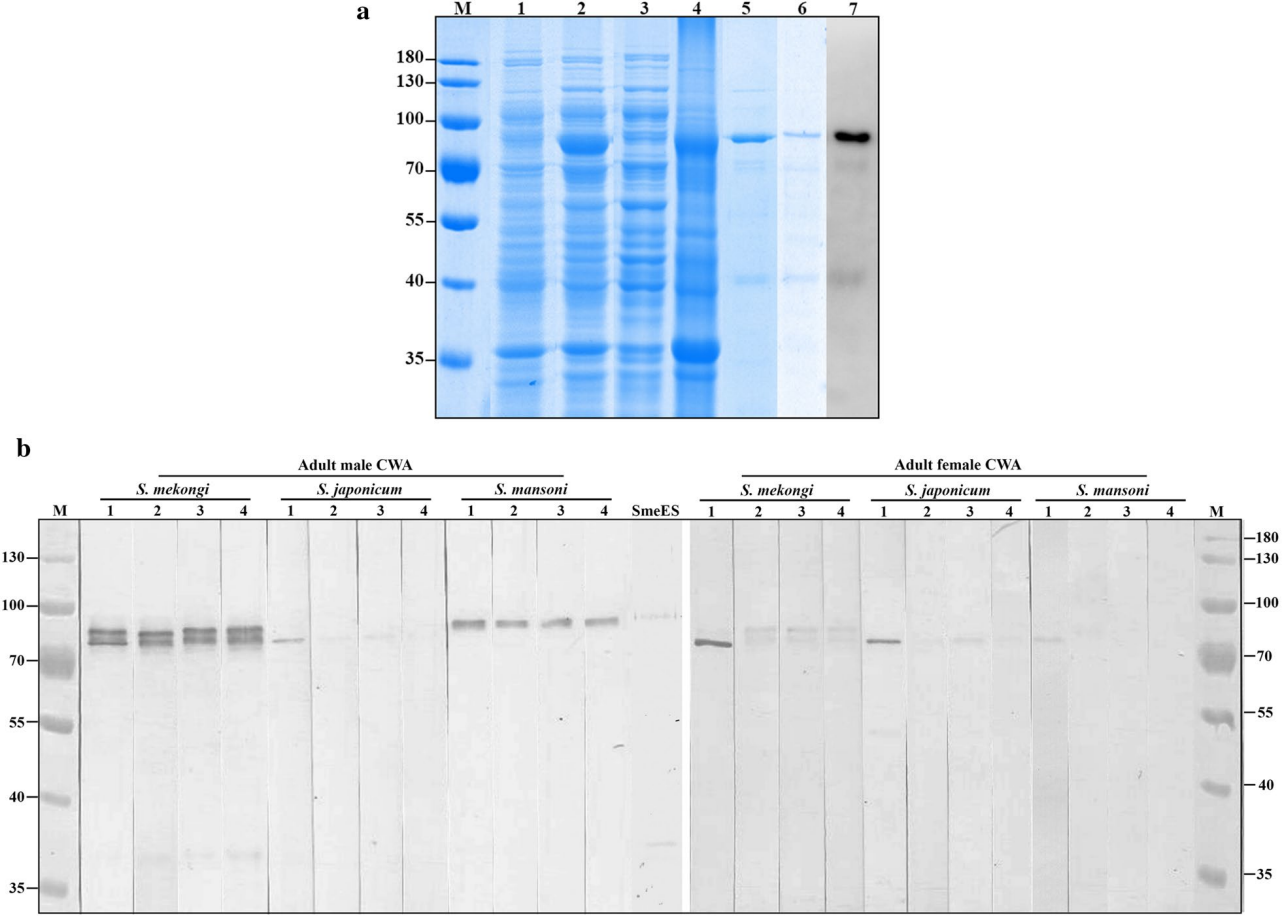
Expression of rSmeCalp1 and detection of native SmeCalp1 in parasite antigens. **a** rSmeCalp1 was successfully expressed in *Escherichia coli*. Lane M: PageRuler Prestained Protein Ladder (Thermo Fisher Scientific); Lane 1: non-induction; Lane 2: IPTG induction; Lane 3: soluble fraction; Lane 4: insoluble fraction; Lane 5: purified rSmeCalp1; Lane 6: refolded rSmeCalp1; Lane 7: western blot analysis of refolded rSmeCalp1 probe with anti-His tag antibody. Arrowhead indicates rSmeCalp1. **b** Western blot analysis detecting native SmeCalp1 in CWA of adult male and female and ES. Lane M: PageRuler Prestained Protein Ladder (Thermo Fisher Scientific Inc.); Lanes 1–4: mice number 1, 2, 3 and 4, respectively. *Abbreviations*: CWA, crude worm antigen; SmeES, *S. mekongi* excretory-secretory product

We induced overexpression of rSmeCalp1 protein in a bacterial expression system using IPTG. The recombinant protein was expressed at a molecular mass of approximately 90 kDa (Fig. 4a). The protein solubility results of rSmeCalp1 suggested that rSmeCalp1 was abundantly expressed in the insoluble fraction. Western blot analysis of rSmeCalp1with mouse anti-His tag antibodies confirmed that rSmeCalp1 was detected only in IPTG-induced bacteria and not without induction (data not shown). rSmeCalp1 was purified under denaturing conditions containing 8 M urea and confirmed by SDS-PAGE and western blot analysis using anti-His tag antibodies. The purified rSmeCalp1 showed a predominant protein band at a molecular mass of approximately 90 kDa and could be detected with anti-His tag antibodies (Fig. 4a). To confirm rSmeCalp1 expression, the gel containing rSmeCalp1 was excised and analyzed by liquid chromatography-tandem mass spectrometry (LC-MS/MS). The MS analysis confirmed that the expressed recombinant protein was SmeCalp1 (Additional file 4: Table S3).

The purified rSmeCalp1 was stepwise dialyzed to remove excess urea and refolded by the dilution method to produce mouse anti-rSmeCalp1 pAb and determine enzymatic activity, respectively. Antibody specificity and antibody titer of mouse anti-rSmeCalp1 pAb were assessed using western blot and ELISA, respectively. rSmeCalp1 induced a highly stimulated antibody response in mice, with a high titer (100,000 to 200,000) in ELISA and specifically reacted with rSmeCalp1 in the western blot (Additional file 5: Figure S2).

### Stage-specific transcription of SmeCalp1

**Fig. 5:**
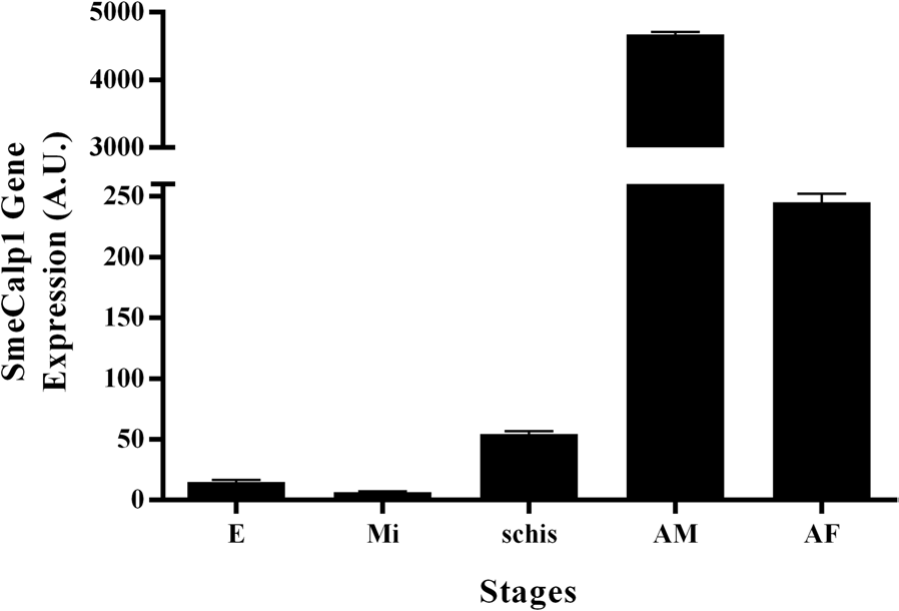
Transcription level of SmeCalp1 in different developmental stages of *Schistosoma mekongi.* Transcription levels of SmeCalp1 in eggs (E), miracidia (Mi), schistosomula (Schis), adult male (AM) and adult female (AF) were determined using SYBR real-time RT-PCR and normalized with expression level of 18S RNA in each stage. The expression level is shown in arbitrary units (A.U.). Error bars show standard deviations. The experiments were performed in triplicate and repeated three times

As mentioned above, SmeCalp1 was predominantly transcribed in adult males over females (Fig. 1a). However, transcription of SmeCalp1 in other developmental stages must be determined to suggest the role of this protein in each developmental stage. Our results indicated that SmeCalp1 was transcribed in all developmental stages (egg, miracidium, schistosomule, adult male and adult female) (Fig. 5). SmeCalp1 was highly expressed in the adult stage, especially in adult males (4700 A.U.), with expression nearly 20 times higher than that in adult females (250 A.U.). The transcription level of SmeCalp1 gene in adult males was approximately 100- to 500-fold higher than that in eggs (16 A.U.), miracidia (7 A.U.) and schistosomula (56 A.U.).

### Detection of native SmeCalp1 in parasite antigens and tissues

Native SmeCalp1 was detected in parasite antigens, CWA and ES using immunoblot analysis reacted with mouse anti-rSmeCalp1 pAb. SmeCalp1 was mainly detected in CWA of both adult male and female parasites. Mouse anti-rSmeCalp1 pAb reacted with SmeCalp1 at different molecular weights (80–90 kDa) in adult male, but reacted with a single molecular weight (80 kDa) in adult female (Fig. 4b). Cross-reactivity of mouse anti-rSmeCalp1 pAb was observed with CWA of *S. japonicum* and *S. mansoni.* SmeCalp1 was also detected in ES product of adult *S. mekongi* (Fig. 4b). Pre-immunized sera did not react with either CWA or ES of the parasite (data not shown).

**Fig. 6:**
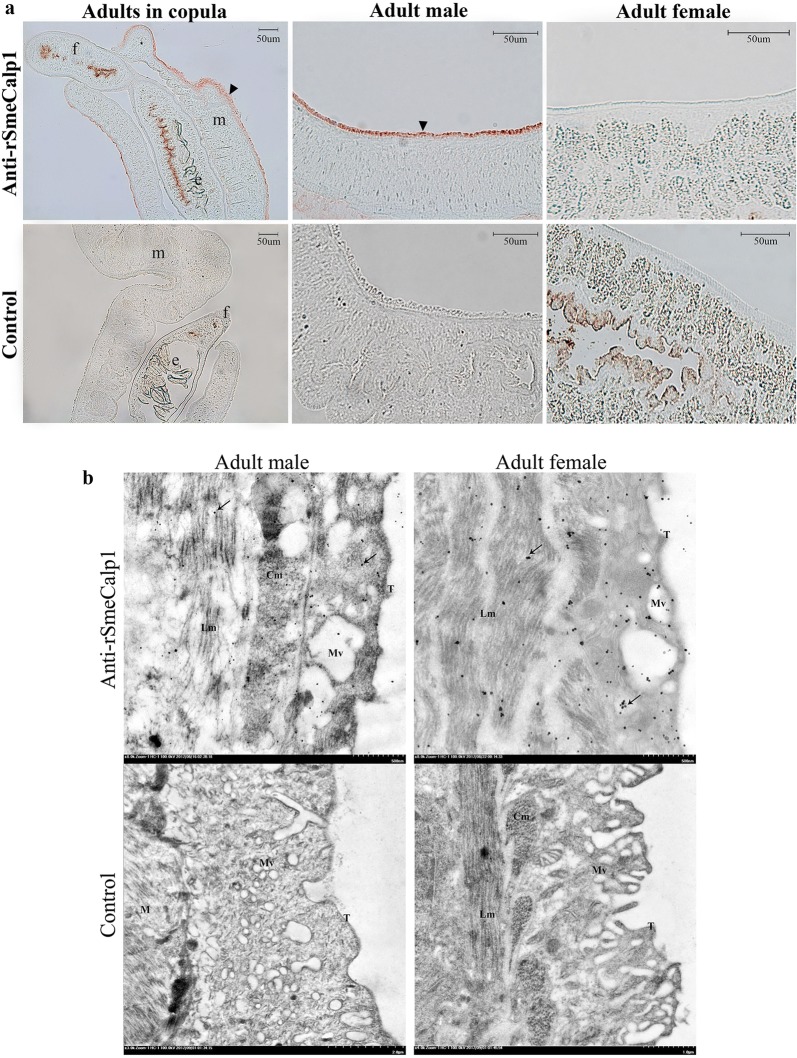
Localization of native SmeCalp1 in *Schistosoma mekongi* adult worm. **a** Immumohistochemical analysis of SmeCalp1 in adult worms. Arrowheads indicate the tegument of *S. mekongi* which reacted with mouse anti-rSmeCalp1 pAb. **b** Electron micrograph of the adult tegument showing immunogold labeling of SmeCalp1. Arrows indicate SmeCalp1 reacted with mouse anti-rSmeCalp1. Left, adult male; right, adult female. *Abbreviations*: m, adult male; f, adult female; e, eggs; T, tegumental surface; M, muscle; Lm, longitudinal muscle layers, Cm, circular muscle layers; Mv, membrane bound vesicle

Immunohistochemistry was performed to localize SmeCalp1 expression in adult *S. mekongi* tissue. After reacting with mouse anti-rSmeCalp1 pAb, SmeCalp1 was predominantly localized on the tegument of the adult male worm, whereas low signal was detected on the tegument of the adult female worm (Fig. 6a). SmeCalp1 was not detected in other organs of either sex (data not shown). Reaction with pre-immunized sera (negative control) was negative. Ultrastructural localization of SmeCalp1 in adult *S. mekongi* was determined using immunogold electron microscopy. SmeCalp1 was localized on the tegumental surface, in membrane-bound vesicles and in circular and longitudinal muscle layers of both sexes (Fig. 6b). Incubation with pre-immune sera showed no immunogold signal in parasite sections.

### Determination of rSmeCalp activity using fluorogenic peptide substrates

**Fig. 7:**
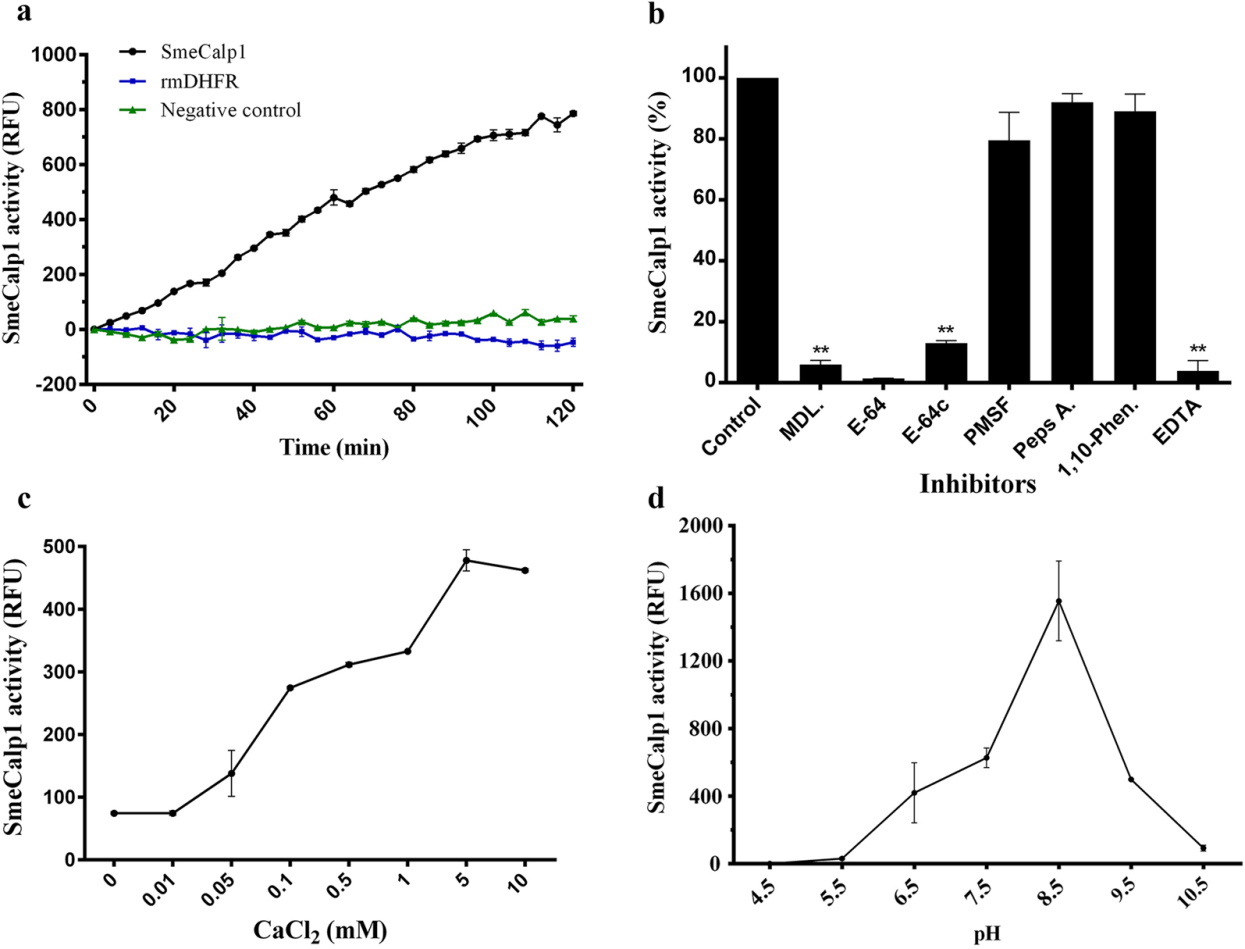
Proteolytic activity of rSmeCalp1. **a** rSmeCalp1 elicited proteolytic activity to hydrolyze the calpain fluorogenic substrate (*N*-succinyl-Leu-Leu-Val-Try-7-AMC), and irrelevant (rmDHFR) and negative controls could not be detected. **b** Proteolytic activity of rSmeCalp1 was inhibited by calpain inhibitors (MDL and E64c), broad cysteine protease inhibitor (E64) and metal chelating agent (EDTA) but not by other protease class inhibitors (PMSF, Peps A and 1,10-Phen). Statistical analysis was performed using Student’s t-test. **P < 0.01. **c** Proteolytic activity of rSmeCalp1 was dependent on concentration of Ca^2+^. **d** A wide pH range for proteolytic activity of rSmeCalp1 was observed (6.5–9.5) with an optimum pH at 8.5. Data are presented as means ± standard deviations. The experiments were performed in triplicate and repeated three times

Refolded rSmeCalp1 was used to assess its proteolytic activity by hydrolyzing the fluorogenic peptide substrate *N*-succinyl-Leu-Leu-Val-Try-7-AMC. After incubation, rSmeCalp1 cleaved the substrate with a gradual increase in fluorescence with incubation time. The irrelevant protein control (rmDHFR) and negative control did not show proteolytic activity (Fig. 7a). To determine class-specific inhibition, rSmeCalp1 was incubated with protease inhibitors of different classes. rSmeCalp1 activity was markedly inhibited by calpain inhibitor MDL28170 (94%), by the broad cysteine protease inhibitors E64 (98%) and E64c (87%), and by the calcium chelating agent EDTA (96%). In contrast, PMSF (serine protease inhibitor), 1,10-phenanthroline (metalloprotease inhibitor) and pepstatin A (aspartic protease inhibitor) did not affect calpain activity of rSmeCalp1 (Fig. 7b).

In previous studies, the activity of calpain was shown to depend on Ca^2+^ concentration [10, 49, 50]. In this regard, we determined the calcium requirement for enzymatic activity by incubating rSmeCalp1 with various concentrations of Ca^2+^. We found that the activity of rSmeCalp1 was dependent on Ca^2+^ concentration, with increased substrate hydrolysis when Ca^2+^ concentration increased. Activity of rSmeCalp1 initially increased at 0.05 mM Ca^2+^ and reached peak activity at 5 mM Ca^2+^ (Fig. 7c). A low level of enzyme activity occurred in the absence of Ca^2+^ or following pretreatment with EDTA (calcium chelating agent; data not shown). In addition to being Ca^2+^ dependent, calpain activity relies on the pH of the environment. A pH-dependence assay revealed that rSmeCalp1 was active over a broad pH range, 6.5 to 9.5, with optimal activity at pH 8.5. Enzymatic activity of rSmeCalp1was inhibited at strong acidic pH (4.5) or strong basic pH (10.5) (Fig. 7d).

### Determination of rSmeCalp activity using biological substrates

**Fig. 8:**
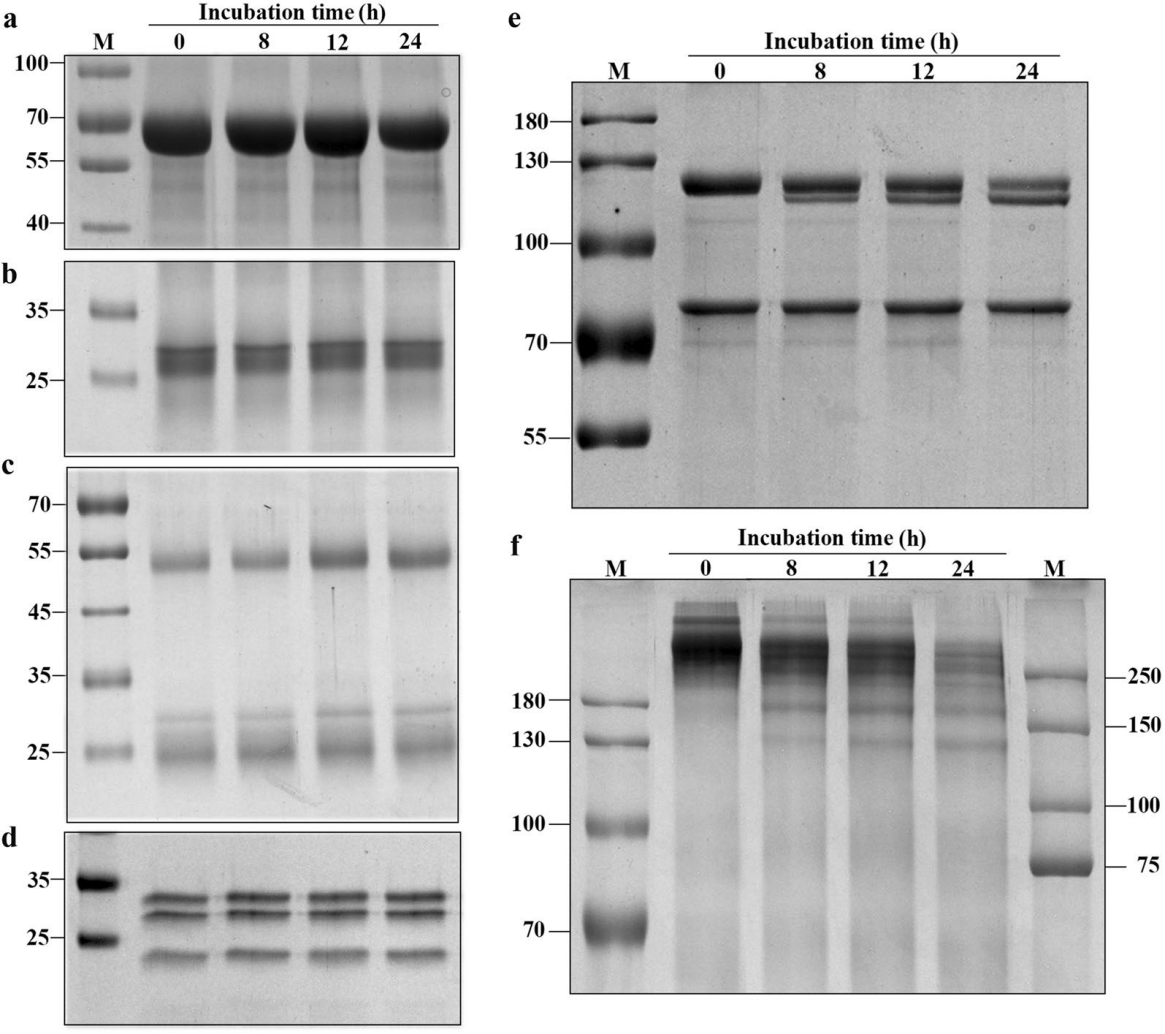
Degradation of biological substrates by rSmeCalp1. SmeCalp1 was incubated with substrates **a** albumin, **b** hemoglobin, **c** IgG, **d** complement C1q, **e** complement C3 and **f** fibronectin at different time points and then subjected to SDS-PAGE. rSmeCalp1 degraded only the α-subunit of complement C3 (115 kDa) (**e**) and fibronectin (**f**), and this activity was inhibited in the presence of cysteine protease inhibitor (E64) (data not shown)

To investigate whether SmeCalp1 can degrade host proteins, we incubated biological substrates, including hemoglobin, immunoglobulin G, albumin, fibronectin and complement components C1q and C3, with rSmeCalp1. The results showed that rSmeCalp1 could degrade the α-chain of C3 from a molecular weight of 115 kDa to a lower mass form (Fig. 8e). C3 consists of two chains, a 115 kDa α-chain and a 75 kDa β-chain. Moreover, rSmeCalp1 degraded fibronectin from a molecular weight of 440 kDa to a ladder-like lower weight (Fig. 8f). The degradation of C3 and fibronectin by rSmeCalp1 was inhibited by E64 inhibitor (data not shown). However, rSmeCalp1 could not degrade other biological substrates, including hemoglobin, immunoglobulin G, albumin and C1q (Fig. 8a–d).

## Discussion

Calpains, calcium-dependent cysteine proteases, are indispensable proteolytic enzymes found in various organisms; they are involved in signal transduction, cell morphogenesis, cytoskeletal remodeling, cell cycle regulation, vesicular trafficking, cell differentiation, apoptosis and necrosis [53–55]. Calpain functions have been explored in several protozoal parasites, including *Plasmodium falciparum* [3, 5, 48, 56, 57], *Toxoplasma gondii* [57], *Cryptosporidium parvum* [58], *Trypanosoma brucei* [59, 60] and *T. cruzi* [61], of which calpains of malaria and trypanosomes have been proposed as drug targets for the development of novel protease inhibitors [62, 63]. In the parasitic helminths, calpains have been identified in nematodes [64], trematodes [65] and cestodes [66], but functions and properties have only been characterized in the genus *Schistosoma*. Until now, calpains have been studied as vaccine candidates in schistosomes, especially in *S. japonicum* [13] and *S. mansoni* [52, 67]. However, there is a lack of information in other human schistosome species including *S. mekongi*, which causes human schistosomiasis in Southeast Asia and poses a health issue for the local population and tourists in the region.

In this study, we determined the molecular characteristics and functions of SmeCalp1 derived from *S. mekongi*. Based on the transcriptome of adult male and female *S. mekongi* [23] and validated by real-time PCR, we demonstrated that SmeCalp1 is a male-biased protease. Moreover, the SmeCalp1 isoform had the highest level of transcription of all isoforms in adult males. A different isoform specificity was found in adult females, in which SmeCalp5 was the most transcribed. Therefore, an effective drug or vaccine based on calpain may require a cocktail combining the predominant isoforms of each sex and developmental stage. The basic property of SmeCalp1 was predicted using bioinformatics; its amino acid sequence indicated that it was a potential membrane protein because a transmembrane helix was detected at the N-terminus. Calpain 1 of *S. mansoni* and *S. japonicum* is not reported to be a transmembrane protein but is located at the tegumental surface of the parasite, which strongly supports a role of calpain 1 in membrane association and host–parasite interaction in schistosomes [9–11]. In addition, calpain of *T. brucei* (CAP5.5) contained a motif for myristoylation and palmitoylation, which are necessary for protein–membrane interaction and high-affinity membrane binding [68]. Comparison of sequence homology between SmeCalp1 and other orthologs suggested that SmeCalp1 has high homology with calpain 1 of *S. japonicum* (Sj-CCalp1), *S. mansoni* (SmCalp1) and *S. haematobium* (ShCalp1). Multiple sequence alignment of SmeCalp1 with orthologs demonstrated that SmeCalp1 contained four conserved domains: N-terminal domain (domain I), protease core domain (domain II), C2-like domain (domain III) and EF-hand domain (domain IV), which are signature structures of classical calpain. Sj-CCalp1 and SmCalp1 are also categorized as classical calpains [51, 69] and show high sequence identity with SmeCalp1, especially Sj-CCalp1. Human calpain 1 (HsCAPN1) has low sequence homology with schistosome calpain 1 and has a shorter N-terminal domain. The protease core domain was the most highly conserved in calpain 1 of trematodes and humans, and it contained the characteristic catalytic triad, Cys-His-Asn, of cysteine protease at the active site [70]. C2 domain-like is also highly conserved in schistosomes and *C. sinensis* but less so in humans. In C2-like domain of schistosome calpain 1, a putative Ca^2+^-binding acidic loop (E-E/D-X-D-D/E-X-D-D/E-D-G-X) was detected, which may be involved in targeting protein to cell membranes, binding of Ca^2+^ and phospholipid, and promoting calpain activation [71–73]. The EF-hand domain of SmeCalp1 is composed of four EF-hand motifs, which were highly conserved with those of other schistosome orthologs. This domain is a well-characterized Ca^2+^ binding domain associated with activation of classical calpains [74].

A high sequence homology and a close relationship presented in phylogenetic tree between SmeCalp1 and other orthologs obtained from schistosomes and *C. sinensis* may indicate similar biochemical and biological roles in members of parasitic trematodes, which could guide further functional analysis. Calpains of parasitic protozoa were remotely separated from and exhibited low sequence homology with those of parasitic helminths, which may suggest that these groups have distinct biological properties and functions. SmCalp1 in *S. mansoni* was expressed on the tegumental surface and in muscle and functioned as a mediator of the surface membrane synthetic process [9]. In contrast, calpain from *P. falciparum* plays an important role in cell cycle progression. After knockdown of calpain gene expression, the parasites were delayed in their ability to transition out of the ring stage and progress to the S-phase [56]. *Trypanosoma brucei* calpain (ClpGM6) is a cytoskeletal protein present in the flagellum with a function in morphology development during complex life cycles [60]. The unrooted phylogenetic tree constructed among calpain isoforms in the genus *Schistosoma* showed that SmeCalp1 belonged to the calpain 1 isoform, which was clearly separated from other isoforms. Until now, several calpain isoforms have been identified in the genus *Schistosoma*, and almost all calpain 1 and some calpain 2 have been functionally characterized and evaluated as drug and vaccine targets [10, 51, 69, 75]. However, other calpain isoforms should be characterized in such terms as biochemistry, stage- and sex-specificity, development, fertilization and fecundity to yield information that will be useful in the prevention and control of schistosomiasis in the future.

We predicted the SmeCalp1 tertiary structure using human m-calpain form II [76] as the template and demonstrated that SmeCalp1 consists of 4 domains (I–IV), a feature of classical calpains. The human domain II was separated into sub-domains PC1 and PC2, which fold into one domain when bound to Ca^2+^ to form the active site of protease [77, 78]. For SmeCalp1, domain II was also predicted to separate into two domains, PC1 and PC2. However, interaction between PC1 and PC2 when bound to Ca^2+^ needs to be explored further using *in silico* simulation and crystallography. Domain III (C2L) of SmeCalp1 contained 8 antiparallel β-strands (jelly-roll fold) as also found in human m-calpain form II. The jelly-roll fold has been reported in tumor necrosis factor (TNF)-α, which this structure facilitates resembling monomer together [76, 79]. Moreover, the C2 domain of various Ca^2+^-regulated proteins, such as protein kinase C isoform, synaptotagmins and phospholipase C, is involved in mechanisms for binding calcium and phospholipids [73, 80, 81]. Domain IV (EF) of SmeCalp1 forms predicted calcium-binding domains that contain a helix-loop-helix topology. In previous studies, binding of Ca^2+^ to ligands in the loop of this domain was shown to stimulate conformational changes in calpain structure leading to biochemical functions [2, 78, 82]. However, the roles of the EF-hand calcium-binding domain and proteolytic function in parasites have not been elucidated and need to be evaluated further. The priority in understanding schistosome calpains is to determine their crystal structure before and after Ca^2+^ binding or when interacting with a specific inhibitor, which will facilitate design of specific calpain inhibitors and *in silico* high-throughput screening for drug discovery and vaccine development.

Full-length SmeCalp1 was successfully expressed as an insoluble protein in a prokaryotic expression system at a molecular size of approximately 90 kDa (predicted size 86 kDa). The slight increase in the size of the recombinant protein results from the inclusion of some amino acids in the multiple cloning site of the pET system and His-tag fusion at the C-terminus, which was detected using anti-His tag antibodies. *Schistosoma japonicum* calpain (Re-P20) encodes a protein of 758 amino acid residues with predicted molecular mass of 86.7 kDa, similar to SmeCalp1, and is expressed as an insoluble protein with molecular mass of approximately 90 kDa when fused with pQE31 [69]. Native SmeCalp1 detected by mouse anti-rSmeCalp1 pAb was present in CWA of adult *S. mekongi*. In adult males, native SmeCalp1 was detected at the same molecular weight as full-length SmeCalp1 (86 kDa) and at a slightly lower molecular weight, which may be the activated form of calpain. In human calpain 1 and calpain 2, autocatalytic cleavage at the N-terminus of the 80 kDa large subunit reduces the size to 78 kDa upon activation of these enzymes [3, 83]. However, the biochemical mechanism of the calpain activation process in the parasitic helminth is currently unknown and needs to be investigated. As well as detecting native SmeCalp1 in CWA of adult male and female *S. mekongi*, mouse anti-rSmeCalp1 pAb also elicited cross-reactivity with CWA of *S. japonicum* and *S. mansoni* in both sexes. This confirms the high homology of SmeCalp1 with Sj-CCalp1 (90.10%) and SmCalp1 (85.35%) (Additional file 3: Figure S1). Moreover, the cross-reaction among these species might imply that calpain 1 could be used as a target for development of a pan-vaccine or pan-chemotherapeutic agent for prevention of broad schistosomiasis in humans or animals. In previous studies, calpain 1 has been evaluated as a vaccine candidate against *S. mansoni* and *S. japonicum*; the results were promising, decreasing worm burden, egg fecundity and related pathology [52, 67, 84]. Evaluation of cross-species protection of a SmCalp1 vaccine (Sm-p80) against *S. japonicum* and *S. haematobium* in a rodent model suggested that the vaccine could significantly reduce the worm burden of *S. japonicum* and *S. haematobium* and stimulate humoral and cellular immune responses [84]. Future studies should evaluate the cross-species prophylactic effect of calpain 1 vaccine derived from other schistosomes including SmeCalp1. In addition to CWA, mouse anti-rSmeCalp1 pAb could detect SmeCalp1 in ES of *S. mekongi*, even though this protein did not contain a signal peptide or transmembrane region. In *S. japonicum*, intracellular calpains were also identified in ES proteome of adult worm and in secretions of cercariae [11, 85]. These findings suggest that secretion of SmeCalp1 into an environment may be facilitated by a non-classical secretory pathway or a vesicle-mediated secretory pathway. Analysis of proteins in exosome-like vesicles derived from *S. mansoni* found that calpain was packed inside, which provided evidence of vesicle-mediated secretion that could play an important role in host–parasite interaction [86].

SmeCalp1 was strongly localized on the tegument of adult male *S. mekongi* but not of the adult female, a finding in agreement with SmeCalp1 being predominantly transcribed in the adult male. In *S. mansoni* and *S. japonicum*, calpains were detected in tegumental proteomes of adult worms [87–89] and predominantly localized at the tegumental surface of adult male worms [9–11]. The role of calpain in the surface membrane synthetic process demonstrated in *S. mansoni* showed that calpain mediated the incorporation of methionine and choline into polypeptides and phospholipids in the apical plasma membrane and overlaying envelop but not in the presence of a calpain inhibitor [9]. In *T. cruzi*, treatment with the calpain inhibitor MDL28170 not only interfered with the differentiation process of the parasite but also significantly reduced binding of epimastigotes to the luminal surface midgut of kissing bugs (*Rhodnius prolixus*) [63]. However, functions of schistosome calpains on host tissue binding, facilitating migration and habitat homing, have not been determined and need further investigation. Ultrastructure localization of SmeCalp1 in *S. mekongi* determined using immunogold-labeling electron microscopy confirmed that SmeCalp1 was localized at the tegumental surface and in the muscle layer of both sexes. Additionally, SmeCalp1 was detected in the membrane-bound vesicles at the apical surface, which supports secretion of this protein by a vesicular secretory pathway. To confirm this proposed process, proteomic analysis of exosome from *S. mekongi* and elucidation of its role in host–parasite interaction are needed.

Proteolytic activity of rSmeCalp1 was associated with Ca^2+^ concentration: activity of rSmeCalp1 gradually increased as Ca^2+^ concentration increased. Calpain is a cytosolic protease produced as an inactive enzyme. When the cellular Ca^2+^ level increases, calpain translocates from the cytosol to the membrane, is activated by autocatalytic hydrolysis at the N-terminus of domain I, and dissociates into large and small subunits. However, there is no information regarding small subunit in schistosomes and requires further investigation. Moreover, binding of calpain with Ca^2+^ induces interaction between subdomains IIa and IIb to form a functional active site, which is necessary for activation [80]. pH is another important factor in calpain activation. In this study, rSmeCalp1 actively hydrolyzed fluorogenic substrate in a pH range from 6.5 to 9.5. The ability to work in a broad pH range may indicate its activity in the diverse environments of a parasite’s life-cycle. In *S. mansoni*, SmCalp1 is highly expressed in different developmental stages, including egg, schistosomulum, adult male and adult female [10]. In *S. japonicum*, Sj-JCalp1 was detected in penetration gland and secretions of cercariae [11]. Activity of rSmeCalp1 was inhibited by calpain inhibitor, broad cysteine protease and EDTA. In *P. falciparum*, calpain inhibitor could inhibit parasite proliferation by suppressing degradation of hemoglobin, which further supports calpain as a potential drug target. Recombinant *Pf*-calpain is heterologously expressed and will be used in high-throughput screening for a highly specific inhibitor against this malaria parasite [48]. Therefore, rSmeCalp1 expressed in this study could have the same advantages as a target in schistosomiasis. As noted previously, SmeCalp1 was abundantly located at the tegumental surface of the adult parasite, which may contribute to nutrient digestion, migration and host immune evasion. In the current study, rSmeCalp1 could not degrade host albumin or hemoglobin but did digest fibronectin. Fibronectin is a high-molecular-weight glycoprotein of the extracellular matrix, which plays an important role in cellular processes, including tissue repair, embryogenesis, blood clotting and cell migration/adhesion [90]. In *S. mansoni*, tegumental calpains including SmCalp1 and SmCalp2 can cleave plasma fibronectin [10], which may suggest roles in regulation of blood clotting that facilitate survival of the parasite. Furthermore, rSmeCalp1 was able to cleave complement component C3 but not C1q. In a previous study, calpain of *Porphyromonas gingivalis* (PgTrp), a periodontal pathogen, could cleave complement components C1q and C3, which may be involved in evading the host immune response by inhibiting the complement system [49]. C3 is the most important and abundant protein in the complement system and is required in both the classical and alternative complement activation pathways [91]. In opportunistic fungal infections, mice lacking C3 were susceptible to *Candida albicans* infection and clearance was delayed [92].

## Conclusions

In this study, we characterized the basic molecular properties and biochemical functions of SmeCalp1, which yielded indispensable evidence on parasite survival and host-parasite interaction. Its localization at the tegumental surface and cleavage of the clotting factor fibronectin and complement component C3 strongly emphasize an important role for SmeCalp1 in host immune evasion and as a drug and vaccine target. The active recombinant protein produced in this study will be subjected to high-throughput screening to identify a specific inhibitor that could be used as an alternative anthelminthic drug. Moreover, we plan to evaluate SmeCalp1 for development of a vaccine against *S. mekongi* with cross-protection against other *Schistosoma* spp.

## Additional files

**Additional file 1: Table S1.** List of primers used for analysis of transcription level by SYBR real-time RT-PCR.

**Additional file 2: Table S2.** Abbreviations and accession numbers of calpain orthologs used in this study.

**Additional file 3: Figure S1.** Structure models of SmeCalp1. **a** Secondary structure of the full-length SmeCalp1 was predicted using the SABLE program. The predicted cysteine (C154), histidine (H313) and asparagine (N337) are highlighted in yellow. Potential *N*-, *O*-glycosylation sites and cysteine residues predicted to form disulfide bonds are marked in blue, green and red, respectively. **b** Tertiary structure was simulated by SwissModel using template crystal structure of human m-calpain form II (PDB ID: 1KFU), which was composed of N-terminal domain, protease core subdomain 1 (PC1), protease core subdomain 2 (PC2), C2-like domain (C2L) and EF-hand domain (EF). The active site cleft is indicated by an arrow.

**Additional file 4: Table S3.** Identification of rSmeCalp1 using liquid chromatography-tandem mass spectrometry (LC-MS/MS).

**Additional file 5: Figure S2.** Western blot analysis of mouse anti-rSmeCalp1 pAb against rSmeCalp1. The antibody response of mice against rSmeCalp1 determined by western blot indicated that rSmeCalp1 were specifically detected by mouse anti-rSmeCalp1 sera. *Key*: M, mouse; −, pre-immunized sera; +, rSmeCalp1immunized sera.

## Data Availability

Data supporting the conclusions of this article are included within the article and its additional files.

## Abbreviations

A.U.: arbitrary unit
AMC: aminomethylcoumarin
C2L: C2 domain-like
cDNA: complementary DNA
CWA: crude worm antigen
DCIP: 2,6-dichlorophenolindophenol
EDTA: ethylenediaminetetraacetic acid
ELISA: enzyme-linked immunosorbent assay
ES: excretory-secretory
IgG: immunoglobulin G
IPTG: isopropyl β-d-1-thiogalactopyranoside
LC-MS/MS: liquid chromatography-tandem mass spectrometry
mRNA: messenger RNA
PMSF: phenylmethylsulfonyl fluoride
rmDHFR: recombinant mouse dihydrofolate reductase
