# Supplementary material for: Molecular characterization and functional analysis of the Schistosoma mekongi Ca2+-dependent cysteine protease (calpain)
Source: Parasit Vectors. 2019 Jul 30;12:383. doi: 10.1186/s13071-019-3639-9 (PMC6668146; doi:10.1186/s13071-019-3639-9)
Supplement: Supplementary file 1 — Additional file 1: Table S1. List of primers used for analysis of transcription level by SYBR real-time RT-PCR. [file 13071_2019_3639_MOESM1_ESM.pdf]

Accession No.	Calpain type	Abbreviation	Primer sequences (5′ → 3′)
MK610444	Calpain1	SmeCalp1	Fw: TTGGAAACGACCACATGAAA<br>Rv: CACCAACATAATCCGGTCCT
MK610445	Calpain2	SmeCalp2	Fw: AGGATTTGTGCCTGGTCAAG<br>Rv: CTTTCAAACGGTCCTGTGGT
MK610446	Calpain4,6,7	SmeCalp4,6,7	Fw: CACTGCGCAGACTAGAAGGT<br>Rv: ATTTCAATGAGGGCAAGCAG
MK610447	Calpain5	SmeCalp5	Fw: AAAATCGCAAGAGTGGGATG<br>Rv: TGGCCAAAATTCCTTAGCAC
MK610448	Calpain7.1	SmeCalp7	Fw: AATGGGCTTAGATCGTGCAG<br>Rv: CCACCAGTACCTCCTTTGGA
MK610449	CalpainB1	SmeCalpB1	Fw: GAACGAAAACGCATTGGTCT<br>Rv: CGTGTAGCACAACGACGACT
