# Supplementary material for: Molecular characterization and functional analysis of the Schistosoma mekongi Ca2+-dependent cysteine protease (calpain)
Source: Parasit Vectors. 2019 Jul 30;12:383. doi: 10.1186/s13071-019-3639-9 (PMC6668146; doi:10.1186/s13071-019-3639-9)
Supplement: Supplementary file 2 — Additional file 2: Table S2. Abbreviations and accession numbers of calpain orthologs used in this study. [file 13071_2019_3639_MOESM2_ESM.pdf]

**Protein name**	**Type**	**Accession no.**	**Species**
**Calpain 1**			
SmeCalp1	Calpain 1	MK610444	*Schistosoma mekongi*
Sj-CCalp1	Calpain 1	BAA74718.1	*S. japonicum* Chinese strain
Sj-JCalp1	Calpain 1	BAA74717.1	*S. japonicum* Japanese strain
SmCalp1.1	Calpain 1	P27730.1	*S. mansoni*
SmCalp1.2	Calpain 1	CCD76462.1	*S. mansoni*
SmCalp1.3	Calpain 1	AAA29858.1	*S. mansoni*
ShCalp1.1	Calpain 1	KGB33810.1	*S. haematobium*
ShCalp1.2	Calpain 1	BAF62290.1	*S. haematobium*
CsCalp	Calpain 1	GAA52477.1	*C. sinensis*
EmCalp	Calpain 1	CDS39654.1	*Echinococcus multilocularis*
EgCalp	Calpain 1	CDS23506.1	*E. granulosus*
HmCalp	Calpain 1	CDS33663.2	*Hymenolepis microstoma*
CeCLP-1	Calpain 1	NP_498741.3	*Caenorhabditis elegans*
AsCalp	Calpain 1	ADY41144.1	*Ascaris suum*
TsCalp	Calpain 1	XP_003374578.1	*Trichinella spiralis*
TcCalp	Calpain 1	KHN79979.1	*Toxocara canis*
PfCalp	Calpain 1	ABR18792.1	*Plasmodium falciparum-3D7*
LmCalp	Calpain 1	XP_001685962.1	*Leishmania major*
TcrCalp	Calpain 1	PBJ68854.1	*Trypanosoma cruzi*
TbCalp	Calpain 1	XP_822445.1	*T. brucei*
TgCalp	Calpain 1	KYF39791.1	*Toxoplasma gondii*
CpCalp	Calpain 1	XP_627039.1	*Cryptosporidium parvum*
HsCANP1	Calpain 1	P07384.1	*Homo sapiens*
GgCalp1	Calpain 1	BAA07230.1	*Gullus gallus*
BbCalp1	Calpain 1	XP_006056550	*Bubalus bubalis*
ClCalp1	Calpain 1	XP_540866.2	*Canis lupus familiaris*
**Calpain 2**			
SmeCalp2	Calpain 2	MK610445	*S. mekongi*
SjCalp2	Calpain 2	ATN96085.1	*S. japonicum*
SmCalp2	Calpain 2	ATN96084.1	*S. mansoni*
**Calpain 4,6,7**			
SmeCalp4,6,7	Calpain 4,6,7	MK610446	*S. mekongi*
SmCalp4,6,7	Calpain 4,6,7	XP_018647856.1	*S. mansoni*
**Calpain 5**			
SmeCalp5	Calpain 5	MK610447	*S. mekongi*
SmCalp5	Calpain 5	CCD74722.1	*S. mansoni*

ShCalp5	Calpain 5	KGB33431.1	*S. haematobium*
**Calpain 7**			
SmeCalp7.1	Calpain 7	MK610448	*S. mekongi*
SmCalp7.1	Calpain 7	CCD59637.1	*S. mansoni*
SmCalp7.2	Calpain 7	CCD59636.1	*S. mansoni*
ShCalp7	Calpain 7	KGB35872.1	*S. haematobium*
**Calpain 9**			
ShCalp9	Calpain 9	KGB32188.1	*S. haematobium*
**Calpain A**			
DmCalpA	CalpainA	NP_477047.1	*Drosophila melanogaster*
IrCalp	CalpainA	JAB75389.1	*Ixodes ricinus*
**Calpain B**			
SmeCalpB1	Calpain B	MK610449	*S. mekongi*
SjCalpB1	Calpain B	CAX73750.1	*S. japonicum*
SjCalpB2	Calpain B	CAX73441.1	*S. japonicum*
SmCalpB1	Calpain B	CCD74749.1	*S. mansoni*
SmCalpB2.1	Calpain B	CCD77023.1	*S. mansoni*
SmCalpB2.2	Calpain B	CCD77022.1	*S. mansoni*
ShCalpB1	Calpain B	KGB40458.1	*S. haematobium*
ShCalpB2	Calpain B	KGB40107.1	*S. haematobium*
**Calpain C**			
SmCalpC	Calpain C	CCD75704.1	*S. mansoni*
