# Supplementary figures and images for: Molecular characterization and functional analysis of the Schistosoma mekongi Ca2+-dependent cysteine protease (calpain)
Source: Parasit Vectors. 2019 Jul 30;12:383. doi: 10.1186/s13071-019-3639-9 (PMC6668146; doi:10.1186/s13071-019-3639-9)

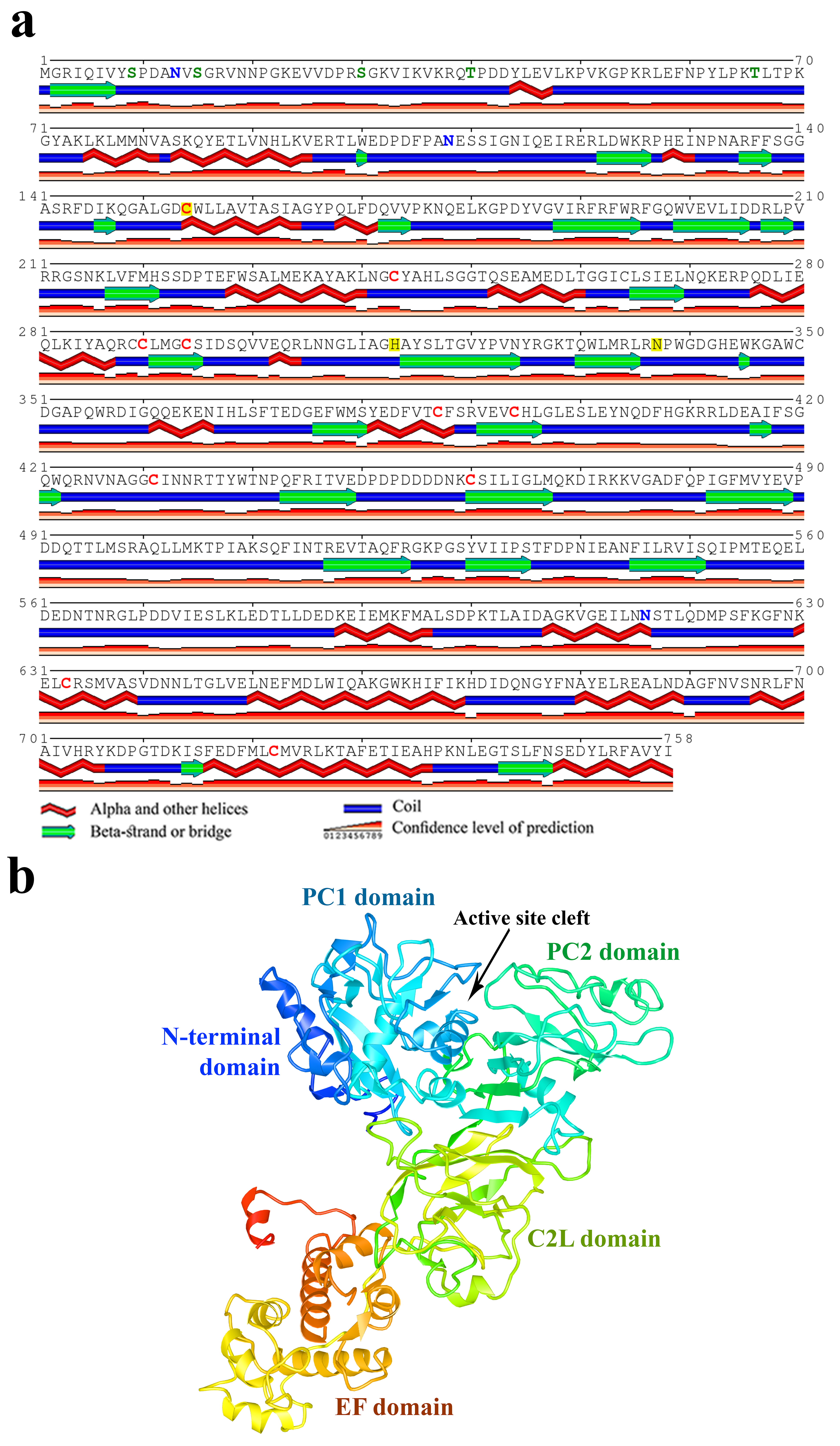

Supplement: Supplementary file 3 — Additional file 3: Figure S1. Structure models of SmeCalp1. a Secondary structure of the full-length SmeCalp1 was predicted using the SABLE program. The predicted cysteine (C154), histidine (H313) and asparagine (N337) are highlighted in yellow. Potential N-, O-glycosylation sites and cysteine residues predicted to form disulfide bonds are marked in blue, green and red, respectively. b Tertiary structure was simulated by SwissModel using template crystal structure of human m-calpain form II (PDB ID: 1KFU), which was composed of N-terminal domain, protease core subdomain 1 (PC1), protease core subdomain 2 (PC2), C2-like domain (C2L) and EF-hand domain (EF). The active site cleft is indicated by an arrow. [file 13071_2019_3639_MOESM3_ESM.tif]

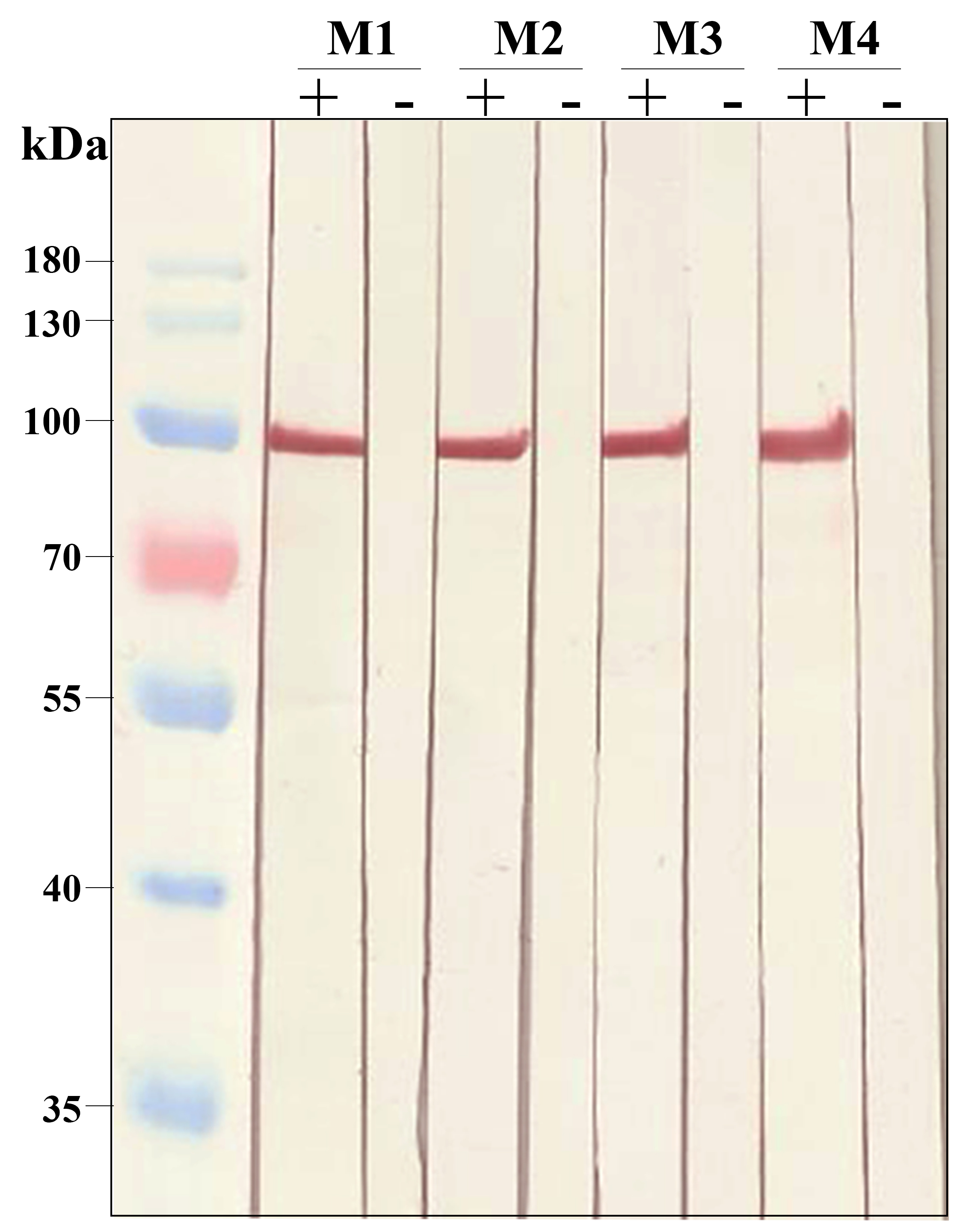

Supplement: Supplementary file 5 — Additional file 5: Figure S2. Western blot analysis of mouse anti-rSmeCalp1 pAb against rSmeCalp1. The antibody response of mice against rSmeCalp1 determined by western blot indicated that rSmeCalp1 were specifically detected by mouse anti-rSmeCalp1 sera. Key: M, mouse; −, pre-immunized sera; +, rSmeCalp1immunized sera. [file 13071_2019_3639_MOESM5_ESM.tif]
